# Influence of Psychological Resilience on Postoperative Outcomes Following Rotator Cuff Repair

**DOI:** 10.7759/cureus.77184

**Published:** 2025-01-09

**Authors:** Mauro E Gracitelli, João Pedro Teixeira Basmage, Leonardo Zanesco, Thais Vasques, Rodrigo A Beraldo, Jorge Assunção, Arnaldo A Ferreira Neto, Eduardo A Malavolta

**Affiliations:** 1 Orthopaedics and Traumatology, Hospital das Clínicas da Faculdade de Medicina da Universidade de São Paulo (HCFMUSP), São Paulo, BRA; 2 Orthopaedics and Traumatology, Instituto Jundiaiense de Ortopedia e Traumatologia (IJOT), Jundiaí, BRA

**Keywords:** arthroscopic surgery, prognostic factors, resilience, rotator cuff tears, the brief resilience scale

## Abstract

Background: Resilience is emerging as a significant factor in orthopedic outcomes, including rotator cuff repair (RCR). However, its influence on postoperative recovery, particularly in different cultural and socioeconomic contexts, remains underexplored.

Methods: This retrospective cohort study included 105 patients who underwent arthroscopic RCR and completed the Brief Resilience Scale (BRS). Clinical outcomes were assessed using the American Shoulder and Elbow Surgeons (ASES) score at 24 months, with secondary evaluations of University of California at Los Angels (UCLA) and Single Assessment Numeric Evaluation (SANE) scores at multiple time points. Resilience levels were categorized as low, normal, or high. Multivariate analysis was performed to identify independent predictors of outcomes, including tear characteristics and resilience.

Results: Patients with high resilience achieved significantly higher ASES scores (91.1 ± 10.6) compared to those with low resilience (58.7 ± 28.8, p = 0.002). Similarly, normal resilience was associated with better outcomes (72.8 ± 26.6, p = 0.043). BRS demonstrated an independent association with outcomes, with each one-point increase in resilience corresponding to a 1.38-point improvement in ASES scores (p = 0.014). Tear characteristics did not independently affect outcomes. Preoperative ASES score emerged as an additional independent predictor (p < 0.001). Socioeconomic context, as inferred by healthcare type, and type of surgery also appear to be relevant factors. Improvements in clinical scores were observed over time across the cohort, but no significant differences were noted between 12-month and 24-month evaluations.

Conclusion: At 24 months postoperatively, resilience significantly influenced clinical outcomes after arthroscopic RCR, with higher BRS scores associated with better ASES outcomes. Patients with low resilience demonstrated worse functional recovery compared to those with normal and high resilience.

## Introduction

The prognosis of rotator cuff repair (RCR) is influenced by a multitude of factors, encompassing both clinical criteria and lesion-specific characteristics [1-4]. Variables such as tear size, tendon retraction and muscle fatty infiltration are well-established determinants of surgical outcomes [2].

In recent years, the role of psychological factors in determining the success of orthopedic surgeries has gained increasing attention. Emerging evidence highlights the profound impact of psychological attributes, including resilience, anxiety, and depression, on functional recovery and patient satisfaction following RCR [5-9].

Resilience, defined as the ability to adapt, recover, and thrive in the face of stress and adversity, has emerged as a critical concept in the evaluation of patient-reported outcomes (PROs). Several studies have validated the importance of resilience in orthopedic outcomes, such as those by Levins et al. [10] in shoulder arthroplasty and Tracy et al. [5] in RCR, which demonstrate significant correlations between resilience scores and post-operative recovery metrics. Tools such as the Brief Resilience Scale (BRS) have been instrumental in quantifying this attribute, offering a standardized approach to integrate psychological resilience into preoperative assessments and surgical planning [11-13].

Despite growing evidence on the role of resilience in orthopedic surgical outcomes, significant gaps remain. Most studies on resilience have been conducted in Western populations, limiting their applicability to different cultural contexts. Latin American populations possess unique social and emotional characteristics that may influence recovery dynamics, making it essential to evaluate the applicability of the BRS in this setting [12,14]. Additionally, the interplay between resilience, shoulder function, and rotator cuff tear patterns remains unclear, particularly in cases involving larger or more complex tears.

This study aims to address these gaps, offering insights into the role of resilience in predicting outcomes and its interaction with clinical and lesion-specific factors. The primary objective of this study was to evaluate the influence of resilience, as measured by the BRS, on the American Shoulder and Elbow Surgeons (ASES) score at 24 months postoperatively. Secondary objectives included assessing the impact of BRS on other functional scores, such as the University of California at Los Angels (UCLA) Shoulder Rating Scale and the Single Assessment Numeric Evaluation (SANE), across multiple time points (preoperative, six months, 12 months, and 24 months), as well as the ASES score at these time points. Additionally, the study aimed to identify other factors that could influence surgical outcomes within our population, including lesion characteristics and socioeconomic factors inferred from the type of healthcare service used (public vs. private). We hypothesize that higher resilience, as measured by the BRS, is associated with better postoperative outcomes.

## Materials and methods

This was a retrospective cohort study with prospectively collected data conducted at the Shoulder and Elbow Group of the Institute of Orthopedics and Traumatology, Shoulder and Elbow Group of the Institute of Orthopedics and Traumatology, Hospital das Clínicas da Faculdade de Medicina da Universidade de São Paulo (HCFMUSP), located in São Paulo, Brazil. The BRS was assessed cross-sectionally at a single time point. The study was approved by the Institutional Ethical Committee of HCFMUSP (approval number: 2.778.930, dated July 20, 2018). The study included patients undergoing rotator cuff surgeries between March 2019 and August 2022. Surgeries were performed by six surgeons from the same institution, all specialized in shoulder surgery, with 15-30 years of experience. Patients were included from both the surgeons' private practices and the public hospital setting.

Eligibility criteria

Patients who underwent arthroscopic repair of rotator cuff tears during the study period were evaluated for inclusion. There were no restrictions on patient age, and all included individuals had demonstrated failure of conservative treatment prior to surgery. Exclusion criteria included: inability to complete the BRS, postoperative follow-up of less than 24 months, and missing preoperative evaluations and the presence of rotator cuff arthropathy.

Outcomes

The primary outcome measure was the ASES score at 24 months postoperatively. Additionally, ASES scores were evaluated at other time points, including preoperatively, and at six and 12 months postoperatively. Secondary outcome measures included the UCLA Shoulder Rating Scale and the SANE score, assessed preoperatively, and at six, 12, and 24 months postoperatively.

The BRS was administered cross-sectionally to all patients. The BRS is a validated six-item questionnaire designed to assess an individual's ability to recover from stress and adversity. Each item is rated on a scale from 0 to 5, generating a total score that reflects resilience. For this study, resilience levels were classified based on standard deviations (SD) from the mean. Patients scoring below 1 SD were categorized as having low resilience (LR), while those scoring 1 SD or higher were classified as having high resilience (HR).

Substantial clinical benefit (SCB) represents the threshold of improvement in outcomes perceived as significant by the patient. For this study, we used the validated ASES score threshold of 86.7, as established by Cvetanovich et al. [15], to define the patient acceptable symptom state following RCR. This threshold helps evaluate clinically meaningful results based on patient-reported outcome measures.

Variables analyzed

Patient-related variables included age, sex, involvement of the dominant arm, smoking status, diabetes status, thyroid conditions, arterial hypertension, previous shoulder trauma, previous shoulder infiltration, and employment-related issues. The patient care setting (public hospital or private practice) was also documented.

Tear-related variables assessed intraoperatively included the presence of supraspinatus, infraspinatus, and/or subscapularis tears. Supraspinatus retraction was categorized as partial tear, full-thickness with retraction < 1 cm, retraction from 1 to 3 cm, retraction > 3 cm. Subscapularis was classified as partial tears, full-thickness tear with no retraction, retraction to the glenoid. Infraspinatus retraction was defined as full-thickness with retraction < 1 cm, retraction from 1 to 3 cm, retraction > 3 cm. Surgery-related variables included subscapularis repair, repair of both supraspinatus and infraspinatus, a biceps procedure (none, tenotomy, or tenodesis), and repair type (single-row or double-row). The type of procedure (open or arthroscopic) was also recorded.

Clinical scores were collected by a research assistant who was blinded to the study and not involved in the surgical procedures.

Surgical procedure

All surgeries were conducted under general anesthesia combined with an interscalene block. Patients were positioned either in the beach chair or lateral decubitus position, depending on the surgeon's preference. Conventional arthroscopic portals were used, and the procedure began with a standardized inspection utilizing a 30° arthroscope inserted through the posterior portal. The long head of the biceps (LHB) was examined using a probe to assess for instability, lesions in its structure, or abnormalities at its insertion site.

Management of the LHB was indicated in cases of subluxation, dislocation, partial tears involving more than 50% of its thickness, or when associated with type 2, 3, or 4 superior labral lesions. Tenotomy was preferred in patients aged 60 years or older, whereas tenodesis was typically performed in younger patients. The subscapularis tendon was repaired in cases of full-thickness tears or when partial tears exposed ≥ 5 mm of the tendon footprint. Posterosuperior tears were repaired using either a single-row or double-row technique, based on the surgeon's preference and implant availability. Acromioplasty was performed for patients with Bigliani type 2 or 3 acromions, and distal clavicle resection was carried out in those with symptomatic arthritis. Open repairs were performed using an anterolateral (mini-open) approach for supraspinatus and infraspinatus tears and a deltopectoral approach for subscapularis repairs.

Postoperatively, patients were immobilized in a sling for six weeks. Passive shoulder movements were initiated at four weeks, active movements began after the sling was removed, and strengthening exercises were introduced at 12 weeks.

Sample size calculation

The sample size was determined by convenience, including all patients who underwent surgery during the study period and had their clinical scores (ASES, UCLA, and SANE) fully completed. From this initial group, a secondary selection was made to include only those patients who completed the BRS in a cross-sectional manner.

Statistical analysis

Baseline Clinical and Intraoperative Analysis

Baseline clinical and intraoperative characteristics were analyzed for the entire cohort. Continuous variables were assessed using Student’s t-test for normally distributed data or the Mann-Whitney U test for non-normal distributions. Categorical variables were evaluated using Chi-square tests or Fisher’s exact tests when sample sizes were small. Descriptive statistics included means and standard deviations for continuous variables and frequencies and percentages for categorical variables.

Outcome Comparison Over Time

Clinical scores were compared across multiple time points (preoperative, six months, 12 months, and 24 months) for the entire cohort. Changes over time were analyzed using repeated measures ANOVA, with post hoc Bonferroni corrections applied to account for multiple comparisons. 

Primary Outcome Subgroup Analysis

The primary outcome, ASES score at 24 months, was further analyzed by resilience level. Differences between high and low resilience groups, as well as between normal and low resilience groups, were assessed using t-tests or Mann-Whitney U tests, depending on data normality.

Correlation Analysis

The association between resilience, as measured by the BRS, and ASES score at 24 months was examined using Pearson’s or Spearman’s correlation coefficients, based on the normality of the data distribution. The strength of the correlation was interpreted according to the Landis and Koch classification [16]. Scatterplots were generated to visually illustrate these relationships, and regression lines were included to enhance interpretability.

SCB Threshold Analysis

The proportion of patients achieving the SCB threshold of 86.7 on the 24-month ASES score was calculated and reported as absolute numbers and percentages. Resilience levels, as measured by the BRS, were compared between patients who achieved and those who did not achieve the SCB threshold, using Mann-Whitney U tests to determine statistical significance.

Univariate Analysis

Relationships between independent variables and the 24-month ASES score were evaluated individually. Continuous variables were assessed using Pearson’s or Spearman’s correlation based on data normality, which was tested using the Shapiro-Wilk test. Categorical variables were analyzed using ANOVA or Kruskal-Wallis tests, as appropriate. Bonferroni corrections were applied to adjust for multiple comparisons, with the significance threshold set at alpha = 0.05/n, where n represents the number of variables analyzed.

Multivariate Analysis

Variables with a significance level of p < 0.10 in the univariate analysis were included in a multiple linear regression model to identify independent predictors of the 24-month ASES score. Regression coefficients (beta), 95% confidence intervals (CI), and p-values were reported. Assumptions of the regression model, including normality of residuals and homoscedasticity, were verified using the Shapiro-Wilk test and residual plots.

Software and Statistical Thresholds

All statistical analyses were performed using Python (Python Software Foundation, Wilmington, Delaware, United States), utilizing the statsmodels (https://www.statsmodels.org/stable/index.html) and SciPy (https://scipy.org/) libraries for computations. A p-value of < 0.05 was considered statistically significant unless otherwise specified. Bonferroni corrections were applied to mitigate the risk of type I errors in multiple comparisons. Missing data were handled using the last observation carried forward (LOCF) method.

## Results

Patient flow

In the time frame of this study, 406 RCRs were performed. From this group, 301 cases were excluded due to the following reasons: postoperative follow-up < 24 months, lack of preoperative evaluation, failure to complete the BRS, rotator cuff arthropathy, or other exclusion criteria outlined in the methods section. After applying these criteria, the final sample consisted of 105 patients. All included patients completed the required clinical scores and responded to the BRS in a cross-sectional manner.

BRS categorization

The BRS was assessed at a mean postoperative time of 45.1 ± 12.7 months. The mean BRS score for the study population was 20.1, with a standard deviation of 4.1. Based on the predefined standard deviation thresholds, patients were categorized into three resilience levels: low, normal, and high resilience. A total of 22 patients (21%) were classified as having low resilience, 73 patients (70%) were categorized as having normal resilience, and 10 patients (10%) were identified as having high resilience.

Baseline

The average age of the patients was 58.4 years (± 8.1). The majority of patients were female (59.0%). The right shoulder was affected in 68.6% of cases. Regarding the type of healthcare service, 70.5% of patients were treated in the public system, while 29.5% received care in private settings. These results are summarized in Table 1.

**Table 1:** Baseline demographic and clinical characteristics for all cases and by resilience level Values are presented as the number with the percentage in parentheses, except for age, which is given as mean±SD Continuous variables were assessed using Student’s t-test for normally distributed data or the Mann-Whitney U test for non-normal distributions; Categorical variables were evaluated using Chi-square tests or Fisher’s exact tests when sample sizes were small. P-value of < 0.05 was considered statistically significant.

Characteristics	Overall (n = 105)	Low resiliency (n = 22)	Normal resiliency (n = 73)	High resiliency (n = 10)	P-value
Age (year), mean±SD	58.4 ± 8.1	58.3 ± 7.6	58.2 ± 8.3	60.4 ± 8.1	0.494
Female	62 (59.0%)	13 (59.1%)	44 (60.3%)	5 (50.0%)	0.923
Involvement of the dominant arm	76 (72.4%)	17 (77.3%)	52 (71.2%)	7 (70.0%)	0.681
Smoking	13 (12.4%)	2 (9.1%)	10 (13.7%)	1 (10.0%)	0.984
Diabetes with insulin use	4 (3.8%)	2 (9.1%)	2 (2.7%)	0 (0.0%)	0.379
Hypothyroidism	13 (12.4%)	3 (13.6%)	6 (8.2%)	4 (40.0%)	0.185
Hypertension	41 (39.0%)	10 (45.5%)	27 (37.0%)	4 (40.0%)	> 0.999
Rheumatoid arthritis	7 (6.7%)	3 (13.6%)	4 (5.5%)	0 (0.0%)	0.567
Work-related claims	18 (17.1%)	8 (36.4%)	9 (12.3%)	1 (10.0%)	0.266
Trauma related tear	37 (35.2%)	8 (36.4%)	27 (37.0%)	2 (20.0%)	0.607
Previous shoulder injection	15 (14.3%)	5 (22.7%)	9 (12.3%)	1 (10.0%)	0.714
Previous shoulder surgery	6 (5.7%)	2 (9.1%)	4 (5.5%)	0 (0.0%)	0.844
Public health system	75 (71.4%)	20 (90.9%)	49 (67.1%)	6 (60.0%)	0.060
Private practice	30 (28.6%)	2 (9.1%)	24 (32.9%)	4 (40.0%)	0.060

The majority of patients had a supraspinatus tear (98.1%), with no significant differences observed across resilience categories (p = 0.681). Double-row repair was performed in 13 patients (12.4%), with the majority belonging to the normal resilience group (11 cases, 84.6%) and 2 cases (15.4%) in the high resilience group (p = 0.168). The low and high resilience groups differed significantly regarding the presence of subscapularis tears (45.5% vs. 100%, p = 0.011). The remaining variables showed no statistically significant differences between the groups. These results are summarized in Table 2.

**Table 2:** Intraoperative characteristics of rotator cuff tears and surgical procedure details Values are presented as the number with the percentage in parentheses. Continuous variables were assessed using Student’s t-test for normally distributed data or the Mann-Whitney U test for non-normal distributions; Categorical variables were evaluated using Chi-square tests or Fisher’s exact tests when sample sizes were small. P-value of < 0.05 was considered statistically significant.

Characteristics	Overall (n = 105), n (%)	Low resiliency (n = 22 ), n (%)	Normal resiliency (n = 73), n (%)	High resiliency (n = 10), n (%)	P
Supraspinatus tear	103 (98.1%)	22 (100.0%)	72 (98.6%)	9 (90.0%)	0.681
Supraspinatus tear classification					
Partial tear	17 (16.2%)	3 (13.6%)	14 (19.2%)	0 (0.0%)	0.514
Retraction < 1cm	10 (9.5%)	4 (18.2%)	5 (6.9%)	1 (11.1%)	
Retraction 1-3 cm	44 (41.9%)	7 (31.8%)	33 (45.2%)	4 (40.0%)	
Retraction > 3cm	32 (30.5%)	8 (36.4%)	20 (27.4%)	4 (40.0%)	
Infraspinatus tear	34 (32.4%)	9 (40.9%)	21 (28.8%)	4 (40.0%)	> 0.999
Infraspinatus tear classification					
Retraction < 1cm	14 (13.3%)	4 (18.2%)	8 (11.0%)	2 (20.0%)	0.719
Retraction 1-3 cm	12 (11.4%)	3 (13.6%)	7 (9.6%)	2 (20.0%)	
Retraction > 3cm	8 (7.6%)	2 (9.1%)	6 (8.2%)	0 (0.0%)	
Subscapularis tear	60 (57.1%)	10 (45.5%)	40 (54.8%)	10 (100.0%)	0.011
Subscapularis tear classification					
No Retraction	44 (41.9%)	7 (31.8%)	30 (41.1%)	7 (70.0%)	0.130
With Retraction	15 (14.3%)	3 (13.6%)	9 (12.3%)	3 (30.0%)	
Retracted to the Glenoid	1 (1.0%)	0 (0.0%)	1 (1.4%)	0 (0.0%)	
Type of surgery					
Open	18 (17.1%)	4 (18.2%)	13 (17.8%)	1 (10.0%)	0.948
Arthroscopic	87 (82.9%)	18 (81.8%)	60 (82.2%)	9 (90.0%)	
Type of repair					
Single-row	92 (87.6%)	22 (100.0%)	62 (84.9%)	8 (80.0%)	0.168
Double-row	13 (12.4%)	0 (0.0%)	11 (15.1%)	2 (20.0%)	
Biceps management					
No procedure	55 (52.4%)	11 (50.0%)	39 (53.4%)	5 (50.0%)	0.256
Tenotomy	22 (21.0%)	4 (18.2%)	16 (21.9%)	2 (20.0%)	
Tenodesis	28 (26.7%)	7 (31.8%)	18 (24.7%)	3 (30.0%)	

Outcome comparison over time

The clinical scores showed significant improvement over time for the entire cohort. The ASES score improved from a mean of 36.0 ± 20.7 preoperatively to 66.6 ± 25.2 at six months, 72.0 ± 25.2 at 12 months, and 71.6 ± 27.2 at 24 months (p < 0.001). Post hoc comparisons revealed significant improvements between the preoperative and all postoperative time points (p < 0.001). However, no significant difference was observed between 12 months and 24 months (p = 0.806).

For the secondary outcomes, the UCLA score increased from 16.1 ± 5.6 preoperatively to 24.7 ± 7.4 at six months, 27.2 ± 7.4 at 12 months, and 28.2 ± 8.6 at 24 months (p < 0.001). Similarly, the SANE score improved from 73.3 ± 21.4 preoperatively to 81.3 ± 20.3 at 12 months, reaching 79.5 ± 22.7 at 24 months (p < 0.001). Post hoc comparisons indicated significant differences across most time points for both UCLA and SANE scores, with the exception of 12 months versus 24 months for SANE (p = 0.289). These results are summarized in Table 3.

**Table 3:** Comparison of baseline and postoperative scores for overall cohort Changes over time were analyzed using repeated measures ANOVA. P-value of < 0.05 was considered statistically significant ASES: American Shoulder and Elbow Surgeons score; UCLA: University of California at Los Angels Shoulder Rating Scale; SANE: Single Assessment Numeric Evaluation

Primary outcome		Overall (n = 105), mean±SD	p-value
ASES			
	Preoperative	36.0 ± 20.7	< 0.001
	At six months	66.6 ± 25.2	
	At 12 months	72.0 ± 25.2	
	At two years	71.6 ± 27.2	
Secondary outcomes			
UCLA			
	Preop	16.1 ± 5.6	< 0.001
	At six months	24.7 ± 7.4	
	At twelve months	27.2 ± 7.4	
	At two years	28.2 ± 8.6	
SANE			
	Preop	50,8 ± 24,0	< 0.001
	At six months	73.3 ± 21.4	
	At twelve months	81.3 ± 20.3	
	At two years	79.5 ± 22.7	

Primary outcome subgroup analysis

The ASES score at 24 months showed significant differences between resilience groups. Patients with high resilience achieved significantly higher scores (91.1 ± 10.6) compared to those with low resilience (58.7 ± 28.8, p = 0.002, Mann-Whitney U). Similarly, the normal resilience group scored higher at 24 months (72.8 ± 26.6) compared to the low resilience group (p = 0.043). No significant differences were observed for preoperative ASES scores between any resilience groups (p = 0.078 and p = 0.138, respectively). These results are summarized in Table 4.

**Table 4:** Comparison of baseline and 24-month postoperative ASES between low- and high-resiliency patients. * P-value for low resiliency versus normal resiliency: p = 0.002; ** P-value for low resiliency versus high resiliency: p = 0.043; P-value of < 0.05 was considered statistically significant Differences between high and low resilience groups, as well as between normal and low resilience groups, were assessed using t-tests or Mann-Whitney U tests, depending on data normality. ASES: American Shoulder and Elbow Surgeons score

		Low resiliency (n = 22 )	Normal resiliency (n = 73)	High resiliency (n = 10 )
ASES	Preop	29.0 ± 16.6	36.4 ± 19.9	48.1 ± 29.2
ASES	At two years	58.7 ± 28.8	72.8 ± 26.6*	91.1 ± 10.6**

For secondary outcomes, the UCLA score at 24 months was higher in the high resilience group (31.9 ± 3.8) compared to the low resilience group (27.1 ± 13.9, p = 0.057), though this difference approached but did not reach statistical significance. No significant differences were observed for SANE scores at 24 months between any resilience groups (high vs. low: p = 0.534; normal vs. low: p = 0.730). 

Correlation analysis

**Figure 1:**
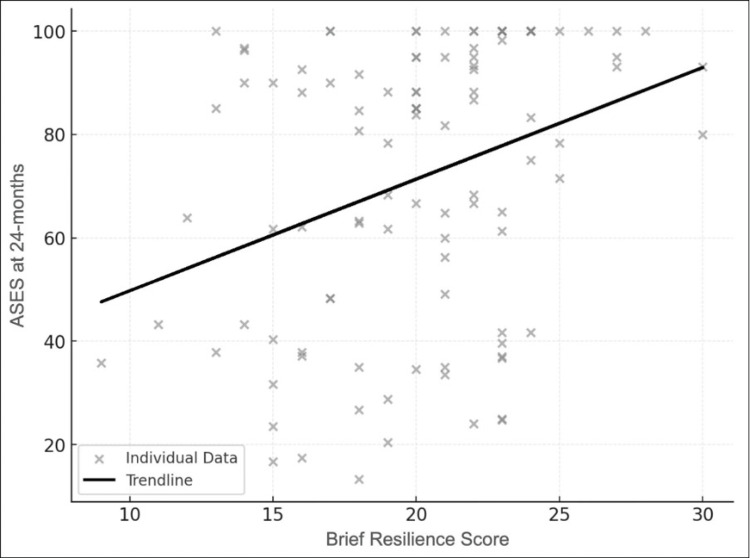
Scatterplot of Brief Resilience Score (BRS) and American Shoulder and Elbow Surgeons (ASES) Score at 24 Months The association between resilience, as measured by the BRS, and ASES score at 24 months was examined using Pearson’s or Spearman’s correlation coefficients, based on the normality of the data distribution.

For the 24-month ASES score, Spearman's correlation coefficient (rho) was 0.342, with a p-value < 0.001, indicating a statistically significant moderate positive correlation. This suggests that higher resilience levels are associated with better functional outcomes at 24 months. In contrast, the preoperative ASES score demonstrated a weak positive correlation with resilience, with a Spearman's correlation coefficient (rho) of 0.111 and a p-value of 0.258. This indicates no statistically significant relationship between resilience and preoperative ASES scores. Scatter plots illustrating these correlation, with regression lines for clarity, are shown in Figure 1.

SCB threshold analysis

The proportion of patients achieving the SCB threshold of 86.7 on the 24-month ASES score was 42.9% (45 patients). Resilience levels, as measured by the BRS, were significantly higher in patients who achieved the SCB (mean BRS 21.2 ± 4.0) compared to those who did not (mean BRS 19.3 ± 3.9), with a p-value of 0.017.

Uni and multivariate analysis

The univariate analysis identified several variables with a potential association with the outcome (p < 0.20), including preoperative ASES score (p < 0.001), BRS (p = 0.0008), type of surgical approach (arthroscopic vs. open) (p < 0.001), age (p = 0.063), type of healthcare service (private vs. public) (p < 0.001), and previous shoulder injection (p = 0.029). These variables were considered for inclusion in the multivariate analysis.

The multiple linear regression analysis identified three independent predictors of the outcome: preoperative ASES score (p < 0.001), BRS (p = 0.014), and the type of surgical approach (arthroscopic vs. open) (p = 0.024). The data are presented in Table 5.

**Table 5:** Multivariate analysis of predictors for ASES score at 24 Months Assumptions of the regression model, including normality of residuals and homoscedasticity, were verified using the Shapiro-Wilk test and residual plots. P-value of < 0.05 was considered statistically significant. ASES: American Shoulder and Elbow Surgeons score; BRS: Brief Resilience Scale; CI: confidence interval

Variable	Coef.	Std. Err.	t	P	95% CI: Lower	95% CI: Upper
Const	0.93	19.65	0.05	0.962	-38.06	39.92
ASES preoperative	0.42	0.12	3.59	0.001	0.19	0.65
BRS	1.38	0.55	2.50	0.014	0.28	2.49
Arthroscopic surgery	13.90	6.08	2.29	0.024	1.84	25.96
Age	0.26	0.27	0.94	0.348	-0.28	0.80
Previous shoulder injection	-8.44	6.33	-1.33	0.185	-21.00	4.12
Private practice	8.20	5.48	1.50	0.137	-2.66	19.07

## Discussion

Our study demonstrated that patients with high resilience achieved an average ASES score of 91.1 points at 24 months postoperatively, compared to 58.7 points in patients with low resilience. This difference of 32.4 points is striking and highlights the profound influence of psychological factors on surgical outcomes. This disparity surpasses the incremental improvements achieved through major technical advancements over the past decades, such as the transition from open to arthroscopic repair [17] and the adoption of double-row techniques [18]. While these innovations have undoubtedly enhanced surgical precision and structural integrity, their impact on functional scores like ASES has been more modest compared to the effect of resilience observed in our study.

Our results align with those of Tracy et al., who reported a mean BRS score of 24.8 in their cohort and demonstrated a significant association between resilience and postoperative outcomes [5]. In their study, patients with high resilience achieved a mean ASES score of 90.2 at 24 months, compared to 68.5 in those with low resilience. In contrast, Hines et al. did not stratify resilience into groups but instead evaluated the mean BRS scores in patients who achieved or did not achieve the SCB threshold [6]. They found no significant difference in resilience scores, which averaged 23.5, regardless of SCB status. However, in our study, this same analysis revealed significant differences. Patients who achieved SCB had a mean BRS score of 21.2 compared to 19.3 in those who did not (p = 0.017). 

Interestingly, the patterns of rotator cuff tears did not independently impact outcomes in our analysis, both in univariate and multivariate models. While this consistency strengthens our findings, the absence of significant associations could be due to our sample size. Previous studies from the same population, with larger cohorts, have demonstrated the prognostic relevance of rotator cuff tear characteristics and surgical procedures. For example, Malavolta et al. identified variables such as fatty degeneration and acromioplasty as predictors of ASES outcomes [1]. Similarly, Beraldo et al. [3] (2024) and Pécora et al. [19] emphasized the importance of lesion type and reparability in influencing clinical and structural outcomes. Additionally, work-related compensation claims, previously identified as influencing outcomes by Assunção et al. [20], did not show a significant effect in our current cohort. These differences highlight the need for larger, multicenter studies to further explore these interactions in diverse populations.

Our univariate analysis identified several variables potentially associated with postoperative ASES scores, including preoperative ASES score, resilience (BRS), surgical approach, type of healthcare service, and previous shoulder injection. However, the multivariate regression confirmed that only preoperative ASES, resilience (BRS), and the type of surgical approach (arthroscopic vs. open) were independent predictors of outcomes at 24 months. Additionally, the identification of preoperative ASES score as an independent predictor reinforces its established role in prognosticating functional outcomes, as seen in prior studies [1,21]. The inclusion of surgical approach as a significant variable in our study suggests differences between arthroscopic and open techniques. However, a recent systematic review did not provide solid evidence to support these differences, as they report no significant disparities in ASES scores or re-rupture rates [22]. 

Limitation and strengths of our study

This study has several limitations that should be considered. The use of the BRS in the postoperative period may introduce recall bias, as patients with better outcomes could inflate their perceived preoperative resilience. The low response rate for the BRS is a limitation of our study, potentially introducing selection bias due to the retrospective design. The absence of mental health evaluations, such as depression or well-being scales, limits the ability to explore their interaction with resilience and functional outcomes. The small number of cases in the high-resilience group also reduces the power to detect subtle differences, although comparisons with the normal-resilience group yielded meaningful findings. Finally, the potential influence of unmeasured confounders cannot be excluded. Larger, multicenter studies with comprehensive prognostic factor analyses are needed to validate and expand upon these results.

Our study presents several strengths. First, it includes a larger sample size compared to the work of Tracy et al. [5], enhancing the statistical power and generalizability of our findings. Second, we incorporated a socioeconomic assessment through the differentiation of healthcare types (public vs. private), providing insights into the interaction between resilience, socioeconomic factors, and surgical outcomes. Finally, our evaluation in a Latin American population offers a unique perspective, addressing cultural variations in resilience and its impact on postoperative recovery. 

The practical implications of our findings emphasize the critical role of mental health in orthopedic outcomes. Recognizing the influence of resilience on recovery allows for better patient counseling, aiding in setting realistic expectations and guiding decisions between conservative and surgical treatments for rotator cuff tears. Low resilience can be flagged using tools like the BRS, enabling early identification of high-risk patients and fostering more tailored, patient-centered care. Integrating psychological support into a multidisciplinary approach has the potential to enhance recovery outcomes. Strategies such as resilience coaching and mindfulness training, proven effective in other settings, could be adapted to orthopedic surgery [23]. Further research is needed to explore the feasibility and cost-effectiveness of such approaches in the perioperative period.

## Conclusions

At 24 months postoperatively, resilience significantly influenced clinical outcomes after arthroscopic RCR, with higher BRS scores associated with better ASES outcomes. Patients with low resilience demonstrated worse functional recovery compared to those with normal and high resilience. 
